# Acoel Flatworms Are Not Platyhelminthes: Evidence from Phylogenomics

**DOI:** 10.1371/journal.pone.0000717

**Published:** 2007-08-08

**Authors:** Hervé Philippe, Henner Brinkmann, Pedro Martinez, Marta Riutort, Jaume Baguñà

**Affiliations:** 1 Canadian Institute for Advanced Research, Centre Robert-Cedergren, Département de Biochimie, Université de Montréal, Montréal, Québec, Canada; 2 Departament de Genètica, Facultat de Biologia, Universitat de Barcelona, Barcelona, Spain; Ecole Normale Supérieure de Lyon, France

## Abstract

Acoel flatworms are small marine worms traditionally considered to belong to the phylum Platyhelminthes. However, molecular phylogenetic analyses suggest that acoels are not members of Platyhelminthes, but are rather extant members of the earliest diverging Bilateria. This result has been called into question, under suspicions of a long branch attraction (LBA) artefact. Here we re-examine this problem through a phylogenomic approach using 68 different protein-coding genes from the acoel *Convoluta pulchra* and 51 metazoan species belonging to 15 different phyla. We employ a mixture model, named CAT, previously found to overcome LBA artefacts where classical models fail. Our results unequivocally show that acoels are not part of the classically defined Platyhelminthes, making the latter polyphyletic. Moreover, they indicate a deuterostome affinity for acoels, potentially as a sister group to all deuterostomes, to Xenoturbellida, to Ambulacraria, or even to chordates. However, the weak support found for most deuterostome nodes, together with the very fast evolutionary rate of the acoel *Convoluta pulchra*, call for more data from slowly evolving acoels (or from its sister-group, the Nemertodermatida) to solve this challenging phylogenetic problem.

## Introduction

Acoelomorph flatworms (Acoela+Nemertodermatida) constitute a small group of bilaterian marine worms that recently came to the limelight. Several morphological similarities suggest that acoelomorphs belong to the Platyhelminthes [1]–[3]; however, these characters are often ill-defined or mere symplesiomorphies [4], leaving the status of the phylum Platyhelminthes unsettled. Molecular phylogenies based on SSU rRNA [5], [6], combined SSU and LSU rRNA [7], myosin II [8] and mitochondrial genomes [9], provide strong statistical support for excluding acoelomorphs from Platyhelminthes. Rather, these markers locate them as a sister-group to all remaining Bilateria.

However, because acoelomorphs evolve at a very high rate (except perhaps for myosin II), their basal emergence can be easily explained by a long branch attraction (LBA) [10] artefact triggered by the distantly related outgroup (Cnidaria). Although careful approaches (e.g. selection of the slowest evolving rRNA sequence among 18 acoels [5]) have been used in an attempt to avoid LBA artefacts, the position of acoelomorphs remains unsettled. In particular, acoelomorphs are often considered as secondarily simplified organisms [11], [12], potentially explaining their fast evolutionary rate. However, finding acoelomorphs at or close to the base of Bilateria, instead of belonging to Platyhelminthes, would allow to polarize several key bilaterian characters including the brain, the coelom, the nephridium, and the possession of a primary larval stage.

We therefore decided to reanalyse this important question by applying the powerful simultaneous analysis of multiple orthologous nuclear protein-coding genes [13] and using up-to-date tree reconstruction methods in order to enhance the phylogenetic signal [14].

## Results and Discussion

We sequenced 2304 ESTs from the acoel *Convoluta pulchra*, a number that has been suggested to provide a sufficient amount of homologous positions [15]. A total of 68 different protein-coding genes was unequivocally assigned to a dataset of conserved single copy genes previously cloned from other multicellular animals and related taxa [14], [16]. A rich taxon sampling of 51 species containing the main bilaterian lineages (Deuterostomia, Ecdysozoa and Lophotrochozoa) and several close and slow-evolving outgroup species (four Cnidaria, three Porifera, three Choanoflagellata and three Ichthyosporea) was selected to reduce as much as possible potential LBA artefacts. After the removal of ambiguously aligned positions and portions that have not been sequenced in *Convoluta*, an alignment of 11,959 positions was obtained.

**Figure 1:**
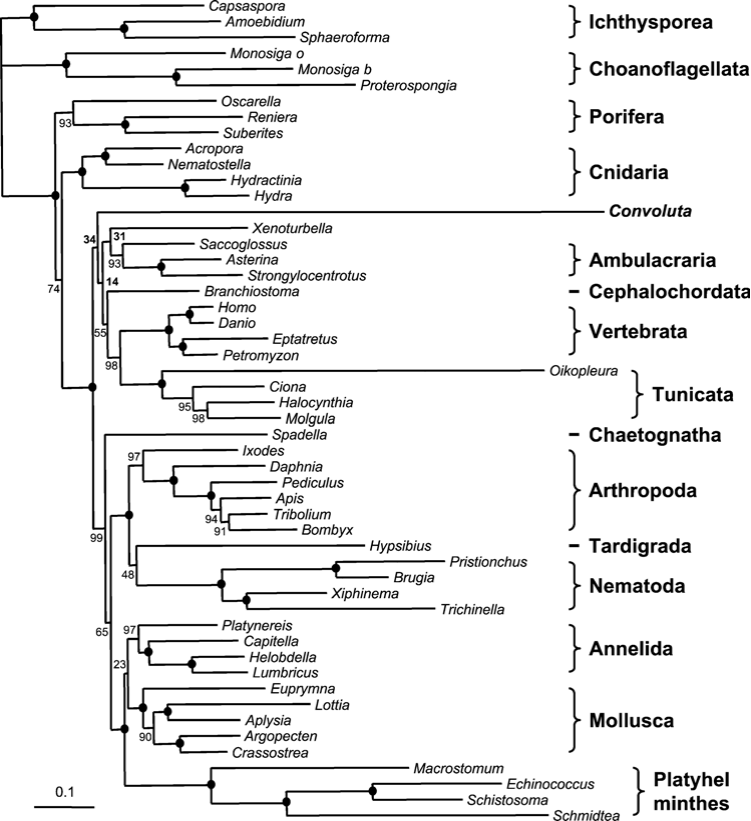
Phylogenetic analysis of genomic data strongly rejects the grouping of *Convoluta* and Platyhelminthes. Bayesian tree obtained from the analysis of 11,959 aligned amino-acid positions with the CAT model. Bootstrap values obtained are indicated when <100%, otherwise a bullet is present on the node. The scale bar indicates the number of changes per site.

The sequences from *Convoluta* are extremely divergent, which make its placement likely to be subject to the LBA artefact. Indeed, when parsimony, a method highly sensitive to this artefact [10], is used, *Convoluta* is invariantly attracted by the longest unbroken branch: the tunicate *Oikopleura* with a Bootstrap Support (BS) of 96% (Figure S1), or the Platyhelminthes (BS = 72%) when *Oikopleura* is excluded (Figure S2). We therefore turned to probabilistic methods that are less sensitive to LBA [17], [18]. Moreover, we used the CAT model [19] because it has been shown to overcome the LBA artefact when other models fail [14], [20]. The resulting tree (Figure 1) is in excellent agreement with current knowledge [12] and with recent large scale analyses [14], [16], [21]–[24]: the monophyly of all animal phyla and of most super-phyla (Bilateria, Protostomia, Ecdysozoa, and Lophotrochozoa) is recovered with strong support (BS>99%). Importantly, *Convoluta* does neither cluster with Platyhelminthes nor with *Oikopleura*, albeit these two groups display the longest branches. Although the precise position of *Convoluta* is not robustly resolved, two highly supported nodes (Protostomia and Lophotrochozoa) separate acoels from Platyhelminthes. The rejection of the traditional morphological hypothesis is therefore very strong.

However, the use of the standard WAG model provides strong support for the grouping of the two long branches platyhelminths and acoels (Figure S3). To further confirm that this grouping is an artefact of both parsimony and the WAG model, we looked for genes that are present in *Convoluta* and in deuterostomes, but absent from protostomes (see supplementary for details). Despite having analysed only 1,664 contigs from *Convoluta* (mainly encoding universal proteins such as ribosomal proteins), we found one gene, the guanidinoacetate N-methyltransferase, that is present in sponges, cnidarians, deuterostomes and *Convoluta*, but absent from all protostomes, except the basal chaetognaths [24]. The tree of the WAG model (Figure S3), which locates acoels as a sister-group to platyhelminths, implies at least three independent losses, whereas the tree of Figure 1 implies a single loss in the protostomes, after the emergence of chaetognaths. The congruence of the tree inferred by the CAT model and of the distribution of guanidinoacetate N-methyltransferase strongly argues that the tree inferred using the WAG model is biased by an LBA artefact, pointing towards a reduced sensitivity of the CAT model to this artefact [14], [20].

Although our phylogenomic tree of Bilateria is generally well resolved, three nodes within protostomes, the positions of chaetognaths, tardigrades and platyhelminths, are poorly supported. More strikingly, most of the relationships among deuterostomes are unsupported. Except for the monophyly of Olfactores [16] and of Ambulacraria [21], the remaining nodes, including deuterostomes and chordates, receive BS below 55%. The very fast evolving *Convoluta* emerges in this part of the tree, as a sister-group to deuterostomes (Figure 1, BS = 14%), to chordates (BS = 33%), or to *Xenoturbella* (BS = 20%). *Convoluta* shows affinity with deuterostomes in 90% of the bootstrap replicates, being basal to Bilateria in only 7% of the replicates and to Protostomia in only 3%. It should be mentioned that the lack of support within deuterostomes is not due to the presence of the long branch of *Convoluta*, since the BS remains low (between 42% and 73%) when *Convoluta* is removed (Figure S4). In consequence, obtaining a reliable placement of *Convoluta* is hampered by two difficulties: (i) its very fast evolutionary rate, and (ii) a location in, or close to, deuterostomes, a region for which the powerful phylogenomic approach only yields poorly supported results [16], [21], [23].

To enhance the phylogenetic signal [25], we removed the outgroup (i.e. non-bilaterian species). Surprisingly, a single topological change occurs (Figure S5): *Xenoturbella* becomes the sister-group to *Convoluta*, albeit with low support (41%). Furthermore, the weak support for the recently proposed *Xenoturbella*+*Ambulacraria* [21] is not due to the presence of *Convoluta* (Figure S6). In summary, our large alignment of 11,959 positions strongly rejects the grouping of acoels with platyhelminths, and more generally protostomes. It favours deuterostome affinity for acoels, potentially as a sister-group to xenoturbellids. However, more data, especially from slowly evolving acoels, are needed to solve this challenging phylogenetic problem.

The rejection of the grouping of acoels with platyhelminths *sensu stricto* (see [4] for a thorough taxonomical discussion) have several morphological implications. First, the lack of protonephridia in acoels, traditionally regarded as derived by the loss from a platyhelminth ancestor bearing them, may now be considered the retention of a primitive condition, a state shared with diploblasts. A similar argument could be applied to the sack-like gut of acoels (and of its sister group, the Nemertodermatida; [26], [27]) which may now be a symplesiomorphy shared with the similar state in diploblasts and independent from the similar sack-like condition of the Platyhelminthes *sensu stricto* within the Lophotrochozoa. In addition, the peculiar duet-spiral cleavage of acoels, also traditionally considered to be derived from the quartet spiral cleavage of other platyhelminths, could now be considered as having originated either from a form of radial or biradial cleavage characteristic of more primitive metazoans. Interestingly, the grouping of Acoelomorpha and Xenoturbellida (Figure S5) has been proposed on the basis of the ultrastructures of epidermal locomotory ciliary rootlets [28] and deserves future studies.

Our results contradict not only the morphological view but also previous molecular phylogenies that strongly support an emergence of acoels at the base of Bilateria [5]–[9], since this position receives here a bootstrap support of only 7%. But, such a basal emergence is expected to be reinforced by a LBA artefact between the long branch of *Convoluta* and the one of the distantly related outgroup. The use of the CAT model, which is less sensitive to LBA [14], [20], partly explains the observed lack of support for a basal emergence, since the latter support increases when the WAG model is used (Figure S7). However, the main reason for the discrepancy with previous molecular studies [5]–[9] is likely that the outgroup is less distantly related in our dataset, thereby reducing the LBA artefact: the distance from cnidarians to the last common ancestor of Bilateria represents 55.5% of the cnidarians/vertebrates distance for our nuclear proteins (Figure 1), but 63.9% for mitochondrial proteins [9] and 73.7% for 18S rRNA [5].

In more conceptual terms, the position of acoels out of the Platyhelminthes should warn us against the naive view that considers some features as ‘lost’, ‘absent’, or ‘reduced’ in clades (e.g. acoels) than might never have had them in the first place. Indeed, the possible position of acoels as a sister-group to Xenoturbellida, another group composed of simple organisms, at the base of deuterostomes leaves open the question of the evolutionary origin of their morphological simplicity.

## Materials and Methods

### Gene cloning

A cDNA library was constructed from adult tissue of the acoel *Convoluta pulchra* using standard methodologies. The cDNAs were cloned in the plasmid vector pSPORT1. A library of several thousand clones was generated, with an average insert size of 1.5 kb. From the generated collection, 2,304 clones were selected and sequenced using the T7 primer. All sequences were grouped and unigenes plus singletons were selected for the phylogenetic analysis.

### Data assembly

Each of the gene alignments used in previous studies [14], [16], [23], [29] was updated with, in addition to *Convoluta* sequences, newly available sequences downloaded from the Trace Archive (http://www.ncbi.nlm.nih.gov/Traces/) and the EST Database (http://www.ncbi.nlm.nih.gov/dbEST/) of GenBank at the National Center for Biotechnology Information (http://www.ncbi.nlm.nih.gov/) using new features of the program ED from the MUST package [30] (see Table S1 for the list of species). Ambiguously aligned regions were automatically detected and removed using the program Gblocks [31] and this selection was manually refined using the program ED.

The concatenation of the 68 genes for which *Convoluta* sequences were available was done by the program ScaFos [32]. ScaFos allows the selection of slowly evolving sequences according to their degree of divergence using ML distances computed under a WAG+F model by Tree-Puzzle [33]. It also permits to reduce the percentage of missing data per taxon by creating chimerical sequences from species belonging to the same predefined taxonomic group (see Table S2). Only genes that are represented for by at least 42 species were considered. The resulting alignment consists of 68 genes, 52 species and 15,554 unambiguously aligned positions. We further removed all positions for which *Convoluta* was not present (due to partial gene sequences), resulting in a final alignment of 11,959 positions. Alignments are available upon request from H.P. (herve.philippe@umontreal.ca).

### Chimerical Operational Taxonomic Units (OTUs)

To increase the amount of information, we created chimerical sequences by using closely related taxa in cases where full length sequences were missing. The list of chimerical species is shown in Table S2. Above the species level OTUs have been named after the most frequently represented species in the data set for that inclusive taxonomic group. The list of the 68 genes used in this study as well as the species missing for each gene is given in Table S3.

### Phylogenetic reconstruction

PhyloBayes analyses were performed with the CAT mixture model, which accounts for across-site heterogeneities in the amino-acid replacement process [19]. Two independent runs were performed with a total length of 15,000 cycles (250 topological moves per cycle) with the same operators as in Lartillot et al. [20]. The first 500 points were discarded as burn-in, and the posterior consensus was computed on the 14,500 remaining trees. Bootstrap proportions were obtained after 100 pseudo-replicates generated with SeqBoot [34]. For computation time reasons, we performed only 2,000 cycles. We verified that 2,000 cycles gave virtually identical results than 14,500 for the complete dataset. In addition, we used a conservative burn-in value of 1,000 (manual verification of a few replicates indicates that the burn-in is less than 500). Trees were collected after the burn-in and for each replicate the consensus of these 1,000 trees was computed by phylobayes. These 100 consensus trees fed to CONSENSE [34] in order to compute the bootstrap support values for each node.

MP heuristic searches were conducted using Paup* [35] with 10 random additions of species, TBR branch swapping and MAXTREES = 1000. MP bootstrap percentages were obtained after 1,000 replications using the same heuristic search strategy using Paup*.

Likelihood-based tests of alternative topologies were calculated using Consel [36]. The topology obtained with the CAT model was used as a backbone on which all possible positions of *Convoluta* were added, yielding 99 (Figure S3) and 91 trees when Platyhelminthes were discarded (Figure S7). ML branch lengths of alternative topologies were first inferred assuming a concatenated WAG+F+Γ_4_ model using Tree-Puzzle [33], site-wise log-likelihood values were then computed with Codeml [37] and p-values of the different likelihood-based tests were calculated with Consel.

### Blast search for gene signature

We searched for genes that could be informative for the phylogenetic position of *Convoluta*, i.e. present in all animals including *Convoluta* but absent, or very divergent, from Platyhelminthes. Given the lack of complete genomes from Lophotrochozoa, we looked for genes absent in protostomes. We used five complete genomes from deuterostomes (*Xenopus tropicalis*, *Homo sapiens*, *Takifugu rubripes*, *Ciona intestinalis*, and *Strongylocentrotus purpuratus*) and four from protostomes (*Caenorhabditis elegans*, *Apis melifera*, *Anopheles gambiae*, and *Drosophila melanogaster*). We run similarity search, using blastx, for the 1664 contigs from *Convoluta* against these genomes and retained 80 sequences that display 10% more similarity to deuterostomes than to protostomes and ten that display 10% more similarity to protostomes than to deuterostomes. A difference of 10% ensures that sequence similarity is a good indicator of phylogenetic affinity (unpublished observation). These 90 sequences were blasted (blastx) against the nr section of NCBI. A manual inspection of the output allowed to eliminate numerous false positives (e.g. genes lost in insects or nematodes, but present in Platyhelminthes or molluscs), yielding 40 genes for a more careful analysis. We then added sequences from additional species (all the species used for our phylogenomic tree). Only one gene was present in *Convoluta* and only in deuterostomes but not in protostomes: the guanidinoacetate N-methyltransferase. We performed a similar analysis to test a basal position of *Convoluta* [5], [8], [9]. We looked for genes that are present in *Convoluta* and in *Nematostella* (http://genome.jgi-psf.org/Nemve1/Nemve1.home.html) but absent from Bilateria. However no genes that would be in favour of a sister-group of *Convoluta* to all Bilateria were found.

## Supporting Information

Table S1 List of the species for which new sequence data have been incorporated in the protein alignments.
(0.01 MB PDF)

Table S2 List of chimerical Operational Taxonomic Units (OTUs).
(0.02 MB PDF)

Table S3 Summary of the occurrence of missing data per taxa in the complete dataset.
(0.02 MB PDF)

Figure S1 Maximum parsimony tree inferred from 11,959 unambiguously aligned amino acid positions with PAUP. The robustness of the phylogenetic inference was estimated by 1000 bootstrap replicates. Nodes supported by 100% bootstrap are denoted by black circles while lower values are given explicitly. The scale bar indicates the number of changes.
(0.02 MB PDF)

Figure S2 Maximum parsimony tree inferred from 11,959 unambiguously aligned amino acid positions without the fast evolving tunicate Oikopleura. The robustness of the phylogenetic inference was estimated by 1000 bootstrap replicates. Nodes supported by 100% bootstrap are denoted by black circles while lower values are given explicitly. The scale bar indicates the number of changes.
(0.02 MB PDF)

Figure S3 Comparison of all possible placements of Convoluta within Holozoa. The possible positions of Convoluta were tested using the program CONSEL (Shimodaira & Hasegawa 2001) on the alignment of 11,959 positions with the WAG+Γ model. Among 99 possible positions, only three were not rejected by the AU test. The number of the topology indicated in second first column is reported on the topology to indicate the position of Convoluta.
(0.02 MB PDF)

Figure S4 Bayesian tree inferred from 11,959 unambiguously aligned amino acid positions without Convoluta using the CAT model. The robustness of the phylogenetic inference was estimated by 100 bootstrap replicates. Nodes supported by bootstrap values of 100% are denoted by black circles while lower values are given explicitly. The scale bar indicates the number of changes per site.
(0.02 MB PDF)

Figure S5 Bayesian tree inferred from 11,959 unambiguously aligned amino acid positions without the outgroup (Ichthyosporea, Choanoflagellata, Porifera and Cnidaria) using the CAT model. The robustness of the phylogenetic inference was estimated by 100 bootstrap replicates. Nodes supported by bootstrap values of 100% are denoted by black circles while lower values are given explicitly. The scale bar indicates the number of changes per site.
(0.01 MB PDF)

Figure S6 Bayesian tree inferred from 11,959 unambiguously aligned amino acid positions without Convoluta and the outgroup (Ichthyosporea, Choanoflagellata, Porifera and Cnidaria) using the CAT model. The robustness of the phylogenetic inference was estimated through 100 bootstrap replicates. Nodes supported by bootstrap values of 100% are denoted by black circles while lower values are given explicitly. The scale bar indicates the number of changes per site.
(0.01 MB PDF)

Figure S7 Comparison of all possible placements of Convoluta within Holozoa when Platyhelminthes are removed. The possible positions of Convoluta were tested using the program CONSEL (Shimodaira & Hasegawa 2001) on the alignment of 11,959 positions with the WAG+Γ model. Among 91 possible positions, six were not rejected by the AU test. The number of the topology indicated in the second column is reported on the topology to indicate the position of Convoluta.
(0.02 MB PDF)
