# Supplementary material for: Acoel Flatworms Are Not Platyhelminthes: Evidence from Phylogenomics
Source: PLoS One. 2007 Aug 8;2(8):e717. doi: 10.1371/journal.pone.0000717 (PMC1933604; doi:10.1371/journal.pone.0000717)
Supplement: Table S1 — List of the species for which new sequence data have been incorporated in the protein alignments. (0.01 MB PDF) [file pone.0000717.s001.pdf]

Table S1. List of the species for which new sequence data have been incorporated in the protein alignments:

Amoebidium parasiticum
Sphaeroforma arctica
Capsaspora owczarzaki
Monosiga brevicollis
Oscarella carmela
Reniera sp.
Suberites domuncula
Nematostella vectensis
Hydra magnipapillata
Hydra vulgaris
Hydractinia echinata
Podocoryne carnea
Acropora millepora
Acropora palmata
Montastraea faveolata
Paracentrotus lividus
Asterina pectinifera
Solaster stimpsonii
Saccoglossus kowalevskii
Xenoturbella bocki
Molgula tectiformis
Halocynthia roretzi
Petromyzon marinus
Biomphalaria glabrata
Aplysia californica
Lymnaea stagnalis
Haliotis discos
Crassostrea virginica
Macrostomum lignano
Dugesia ryukyuensis
Dugesia japonica
Ixodes scapularis
Daphnia pulex
Daphnia magna
Pediculus humanus
