# Supplementary material for: Acoel Flatworms Are Not Platyhelminthes: Evidence from Phylogenomics
Source: PLoS One. 2007 Aug 8;2(8):e717. doi: 10.1371/journal.pone.0000717 (PMC1933604; doi:10.1371/journal.pone.0000717)
Supplement: Table S2 — List of chimerical Operational Taxonomic Units (OTUs). (0.02 MB PDF) [file pone.0000717.s002.pdf]

Table S2. List of chimerical Operational Taxonomic Units (OTUs)

Convoluta: ***Convoluta pulchra****, Convoluta roscoffensis*
Hydra: ***Hydra magnipapillata****, Hydra vulgaris*
Acropora: ***Acropora millepora****, Acropora palmata, Montastraea faveolata*
Hydractinia: ***Hydractinia echinata****, Podocoryne carnea*
Suberites: ***Suberites domuncula****, Suberites fuscus*
Homo: ***Homo sapiens****, Mus musculus, Rattus norvegicus, Macaca fascicularis, Bos taurus, Canis familiaris*
Eptatretus: ***Eptatretus burgeri****, Myxine glutinosa*
Petromyzon: ***Petromyzon marinus****, Lethenteron japonicum, Lethenteron reissneri*
Branchiostoma: ***Branchiostoma floridae****, Branchiostoma belcheri, Branchiostoma lanceolatum*
Ciona: ***Ciona intestinalis****, Ciona savignyi*
Strongylocentrotus: ***Strongylocentrotus purpuratus****, Paracentrotus lividus, Hemicentrotus pulcherrimus*
Asterina: ***Asterina pectinifera****, Solaster stimpsonii*
Spadella: ***Spadella cephaloptera****, Flaccisagitta enflata*
Aplysia: ***Aplysia californica****, Biomphalaria glabrata, Lymnaea stagnalis, Haliotis discus*
Crassostrea : ***Crassostrea virginica****, Crassostrea gigas, Mytilus galloprovincialis, Mytilus edulis*
Argopecten: ***Argopecten irradians****, Pecten maximus, Chlamys farreri*
Schmidtea: ***Schmidtea mediterranea****, Dugesia ryukyuensis, Dugesia japonica*
Schistosoma: ***Schistosoma mansoni****, Schistosoma japonicum*
Echinococcus: ***Echinococcus granulosus****, Echinococcus multilocularis*
Helobdella: ***Helobdella robusta****, Haementeria depressa*
Lumbricus: ***Lumbricus rubellus****, Eisenia andrei*
Ixodes: ***Ixodes scapularis****, Ixodes pacificus*
Daphnia: ***Daphnia pulex****, Daphnia magna*
Hypsibius: ***Hypsibius dujardini****, Macrobiotus islandicus, Richtersius coronifer*
Brugia: ***Brugia malayi****, Wuchereria bancrofti, Litomosoides sigmodontis, Dirofilaria immitis, Onchocerca volvulus, Ascaris suum, Toxocara canis*
