# Supplementary material for: Acoel Flatworms Are Not Platyhelminthes: Evidence from Phylogenomics
Source: PLoS One. 2007 Aug 8;2(8):e717. doi: 10.1371/journal.pone.0000717 (PMC1933604; doi:10.1371/journal.pone.0000717)
Supplement: Table S3 — Summary of the occurrence of missing data per taxa in the complete dataset. (0.02 MB PDF) [file pone.0000717.s003.pdf]

Table S3. Summary of the occurrence of missing data per taxa in the complete dataset

gene	arp23	cct-A	cct-B	cct-D	cct-E	cct-N	cct-Z	ef1-EF1	ef2-EF2	grc5	if2g	l12e-A	nsf1-I	nsf1-K	nsf1-M	nsf2-A	psma-B	psma-C	psma-D	psma-F	psmb-M	psmb-N	rpl1	rpl12b	rpl15a	rpl16b	rpl18	rpl19a	rpl2	rpl21	rpl23a	rpl24-A	rpl25	rpl27
#positions	184	516	498	471	511	498	478	418	733	209	447	118	404	379	411	640	209	203	219	202	199	160	213	162	204	173	178	182	247	152	139	115	117	133
Acropora millepora		X												X						X		X	X											
Amoebidium parasiticum	X	X	X		X	X					X	X			X		X		X			X					X	X		X		X		
Apis mellifera																																		
Aplysia californica																																		
Argopecten irradians		X	X	X	X		X				X		X				X		X			X												
Asterina pectinifera											X									X														
Bombyx mori																																		
Branchiostoma floridae																																		
Brugia malayi																																		
Capitella sp.																																		
Capsaspora owczarzaki																X						X												
Ciona intestinalis																																		
Convoluta pulchra																																		
Crassostrea virginica		X		X							X			X						X														
Danio rerio																																		
Daphnia pulex																																		
Echinococcus granulosus	X	X		X		X	X							X		X		X			X			X										
Eptatretus burgeri													X					X																
Euprymna scolopes												X													X									
Halocynthia roretzi			X							X			X	X						X	X		X		X	X					X	X	X	
Helobdella robusta																																		
Homo sapiens																																		
Hydra magnipapillata																																		
Hydractinia echinata														X																				
Hypsibius dujardini			X			X	X				X		X			X					X	X												
Ixodes scapularis																																		
Lottia gigantea																														X				
Lumbricus rubellus			X				X				X							X	X															
Macrostomum lignano											X				X		X	X					X									X		X
Molgula tectiformis																																		
Monosiga brevicollis								X																										
Monosiga ovata							X							X						X														
Nematostella vectensis																																		
Oikopleura dioica																																		
Oscarella carmela											X	X					X	X		X	X				X			X		X	X	X		X
Pediculus humanus																																		
Petromyzon marinus							X																											
Platynereis dumerilii		X				X		X		X		X	X				X	X	X		X	X		X		X	X			X		X	X	X
Pristionchus pacificus																																		
Proterospongia sp.	X			X	X	X	X	X			X	X	X				X		X		X				X				X	X			X	
Reniera sp.																																		
Saccoglossus kowalevskii																																		
Schistosoma mansoni																																		
Schmidtea mediterranea																																		
Spadella cephaloptera	X	X	X	X	X	X	X				X		X		X	X	X		X		X													
Sphaeroforma arctica		X						X						X	X			X		X														
Strongylocentrotus purpuratus																																		
Suberites domuncula	X	X	X	X	X	X	X	X	X				X	X	X	X	X	X	X	X	X	X												
Tribolium castaneum																																		
Trichinella spiralis																																		
Xenoturbella bocki		X			X	X							X	X		X	X	X	X	X	X	X					X			X		X		
Xiphinema index	X												X	X	X																			

Table S3. Followed

gene	rpl3	rpl30	rpl31	rpl32	rpl33a	rpl35	rpl37a	rpl42	rpl4B	rpl5	rpl6	rpl7-A	rpl9	rpp0	rps1	rps13a	rps14	rps15	rps16	rps17	rps18	rps19	rps2	rps20	rps22a	rps23	rps25	rps27	rps29	rps3	rps4	rps6	rps8	sap40
#positions	370	104	98	123	107	121	75	105	277	238	118	193	166	272	242	150	137	139	136	100	152	129	212	101	130	142	87	82	54	210	252	209	195	206
Acropora millepora		X						X																										
Amoebidium parasiticum						X					X			X						X								X		X				
Apis mellifera																																		
Aplysia californica																																		
Argopecten irradians																																		
Asterina pectinifera							X																											
Bombyx mori																																		
Branchiostoma floridae																																		
Brugia malayi																																		
Capitella sp.																																		
Capsaspora owczarzaki																																		
Ciona intestinalis																																		
Convoluta pulchra																																		
Crassostrea virginica																																		
Danio rerio																																		
Daphnia pulex																																		
Echinococcus granulosus		X																																
Eptatretus burgeri																																		
Euprymna scolopes							X	X												X					X		X	X	X					
Halocynthia roretzi					X	X	X				X	X			X	X	X		X	X	X	X		X	X	X	X	X	X		X			
Helobdella robusta																																		
Homo sapiens																																		
Hydra magnipapillata																																		
Hydractinia echinata																																		
Hypsibius dujardini	X										X												X											
Ixodes scapularis																																		
Lottia gigantea			X																						X				X				X	
Lumbricus rubellus																																		
Macrostomum lignano		X					X	X					X												X		X							
Molgula tectiformis																																		
Monosiga brevicollis																																		
Monosiga ovata																													X					
Nematostella vectensis																																		
Oikopleura dioica																													X					
Oscarella carmela		X	X	X			X	X					X			X		X		X	X	X		X	X	X	X		X			X		
Pediculus humanus																												X						
Petromyzon marinus																																		
Platynereis dumerilii		X	X		X		X	X			X	X	X		X			X	X	X	X	X					X	X	X					X
Pristionchus pacificus																																		
Proterospongia sp.	X	X		X	X	X	X			X												X					X				X			
Reniera sp.																																		
Saccoglossus kowalevskii																																		
Schistosoma mansoni																																		
Schmidtea mediterranea																																		
Spadella cephaloptera									X																									
Sphaeroforma arctica																																		
Strongylocentrotus purpuratus																																		
Suberites domuncula																																		
Tribolium castaneum																																		
Trichinella spiralis																													X					
Xenoturbella bocki		X	X		X		X	X	X					X				X		X								X	X			X		
Xiphinema index		X																						X										

Table S3. Followed

Concatenation	%missing positions	
#positions	15554	11959
Acropora millepora	26	25.2
Amoebidium parasiticum	55	51.2
Apis mellifera	2	1.2
Aplysia californica	14	14
Argopecten irradians	33	26.1
Asterina pectinifera	22	18.9
Bombyx mori	1	0.7
Branchiostoma floridae	1	1
Brugia malayi	11	9.4
Capitella sp.	7	6.8
Capsaspora owczarzaki	27	15.9
Ciona intestinalis	0	0.2
Convoluta pulchra	23	0
Crassostrea virginica	24	21.5
Danio rerio	0	0
Daphnia pulex	1	0.4
Echinococcus granulosus	34	31.6
Eptatretus burgeri	12	9.7
Euprymna scolopes	26	27.6
Halocynthia roretzi	41	40.5
Helobdella robusta	12	10.7
Homo sapiens	0	0
Hydra magnipapillata	0	0
Hydractinia echinata	17	10.9
Hypsibius dujardini	40	29.9
Ixodes scapularis	3	2.8
Lottia gigantea	41	39.7
Lumbricus rubellus	27	21.6
Macrostomum lignano	35	28.8
Molgula tectiformis	1	1.1
Monosiga brevicollis	4	3.7
Monosiga ovata	20	17.9
Nematostella vectensis	0	0
Oikopleura dioica	4	3.6
Oscarella carmela	41	41.7
Pediculus humanus	4	3.1
Petromyzon marinus	7	5.9
Platynereis dumerilii	54	51.1
Pristionchus pacificus	6	4.3
Proterospongia sp.	55	51.3
Reniera sp.	1	0.7
Saccoglossus kowalevskii	10	9.4
Schistosoma mansoni	0	0.1
Schmidtea mediterranea	1	0.8
Spadella cephaloptera	47	39.9
Sphaeroforma arctica	30	22.7
Strongylocentrotus purpuratus	0	0.1
Suberites domuncula	50	41.4
Tribolium castaneum	0	0
Trichinella spiralis	5	5.6
Xenoturbella bocki	51	47.3
Xiphinema index	24	18.7
