# Supplementary material for: Acoel Flatworms Are Not Platyhelminthes: Evidence from Phylogenomics
Source: PLoS One. 2007 Aug 8;2(8):e717. doi: 10.1371/journal.pone.0000717 (PMC1933604; doi:10.1371/journal.pone.0000717)
Supplement: Figure S1 — Maximum parsimony tree inferred from 11,959 unambiguously aligned amino acid positions with PAUP. The robustness of the phylogenetic inference was estimated by 1000 bootstrap replicates. Nodes supported by 100% bootstrap are denoted by black circles while lower values are given explicitly. The scale bar indicates the number of changes. (0.02 MB PDF) [file pone.0000717.s004.pdf]

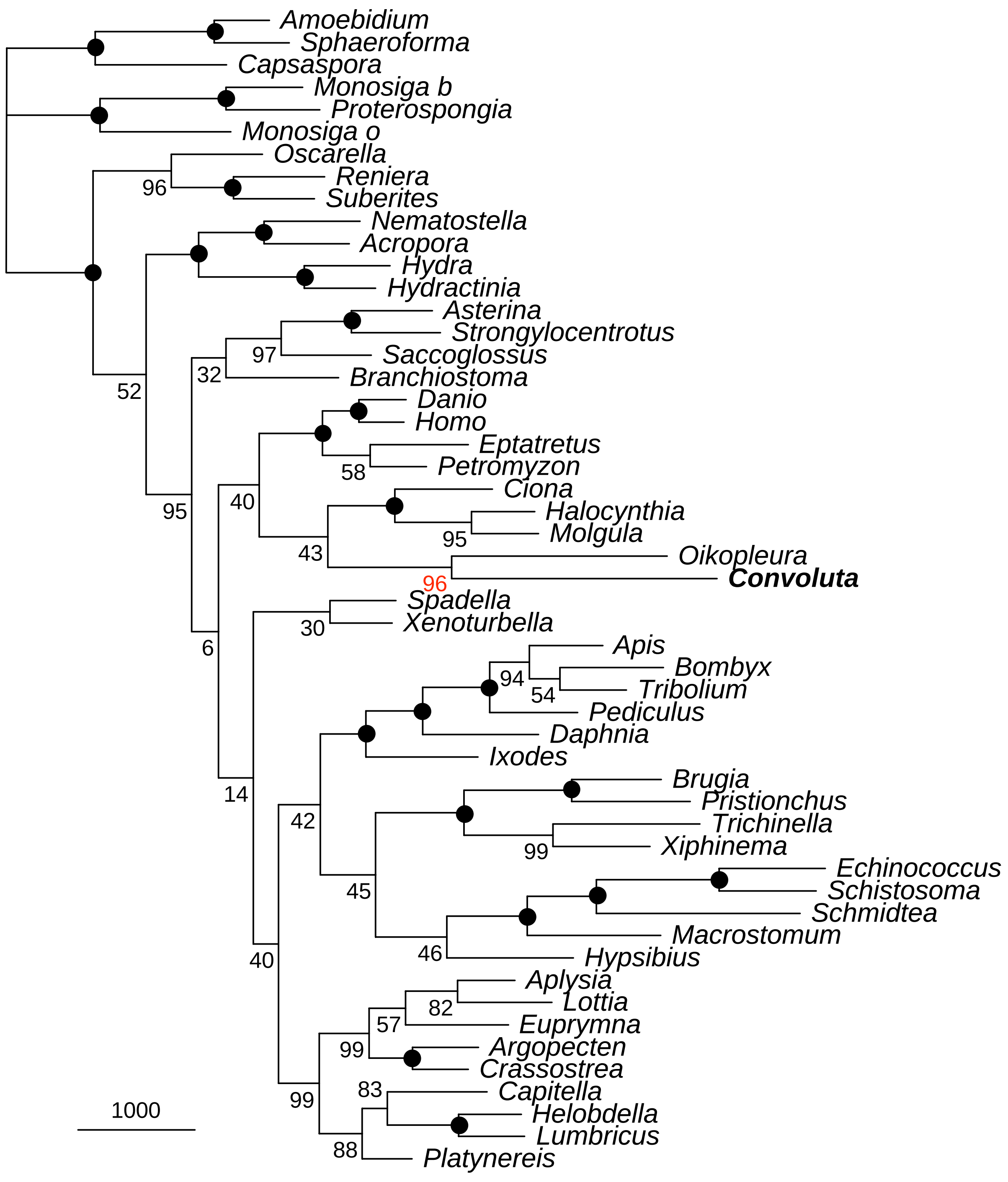

Figure S1: Maximum parsimony tree inferred from 11,959 unambiguously aligned amino acid positions with PAUP. The robustness of the phylogenetic inference was estimated by 1000 bootstrap replicates. Nodes supported by 100% bootstrap are denoted by black circles while lower values are given explicitly. The scale bar indicates the number of changes.
