# Supplementary material for: Acoel Flatworms Are Not Platyhelminthes: Evidence from Phylogenomics
Source: PLoS One. 2007 Aug 8;2(8):e717. doi: 10.1371/journal.pone.0000717 (PMC1933604; doi:10.1371/journal.pone.0000717)
Supplement: Figure S2 — Maximum parsimony tree inferred from 11,959 unambiguously aligned amino acid positions without the fast evolving tunicate Oikopleura. The robustness of the phylogenetic inference was estimated by 1000 bootstrap replicates. Nodes supported by 100% bootstrap are denoted by black circles while lower values are given explicitly. The scale bar indicates the number of changes. (0.02 MB PDF) [file pone.0000717.s005.pdf]

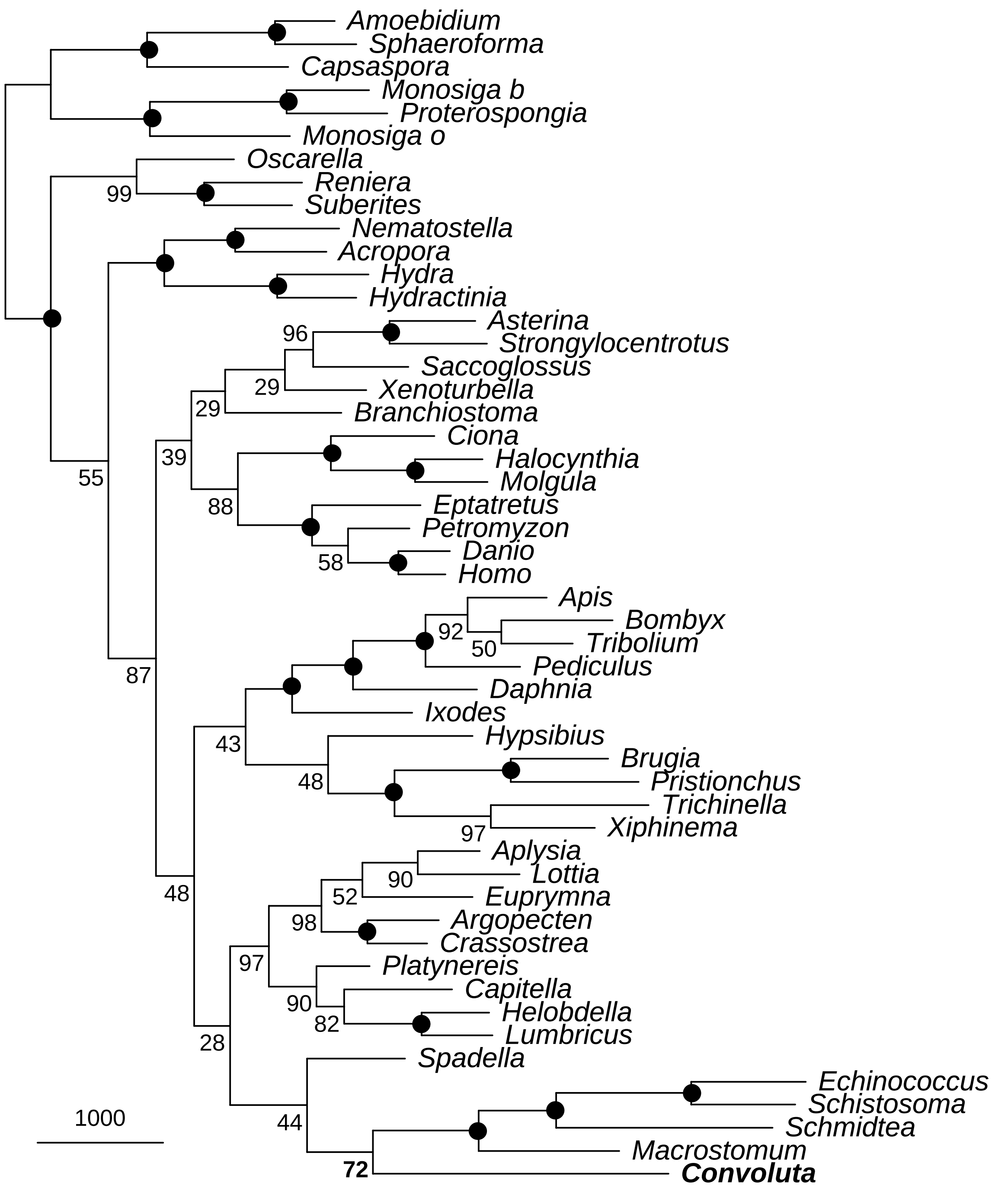

Figure S2: Maximum parsimony tree inferred from 11,959 unambiguously aligned amino acid positions without the fast evolving tunicate *Oikopleura*. The robustness of the phylogenetic inference was estimated by 1000 bootstrap replicates. Nodes supported by 100% bootstrap are denoted by black circles while lower values are given explicitly. The scale bar indicates the number of changes.
