# Supplementary material for: Acoel Flatworms Are Not Platyhelminthes: Evidence from Phylogenomics
Source: PLoS One. 2007 Aug 8;2(8):e717. doi: 10.1371/journal.pone.0000717 (PMC1933604; doi:10.1371/journal.pone.0000717)
Supplement: Figure S3 — Comparison of all possible placements of Convoluta within Holozoa. The possible positions of Convoluta were tested using the program CONSEL (Shimodaira & Hasegawa 2001) on the alignment of 11,959 positions with the WAG+Γ model. Among 99 possible positions, only three were not rejected by the AU test. The number of the topology indicated in second first column is reported on the topology to indicate the position of Convoluta. (0.02 MB PDF) [file pone.0000717.s006.pdf]

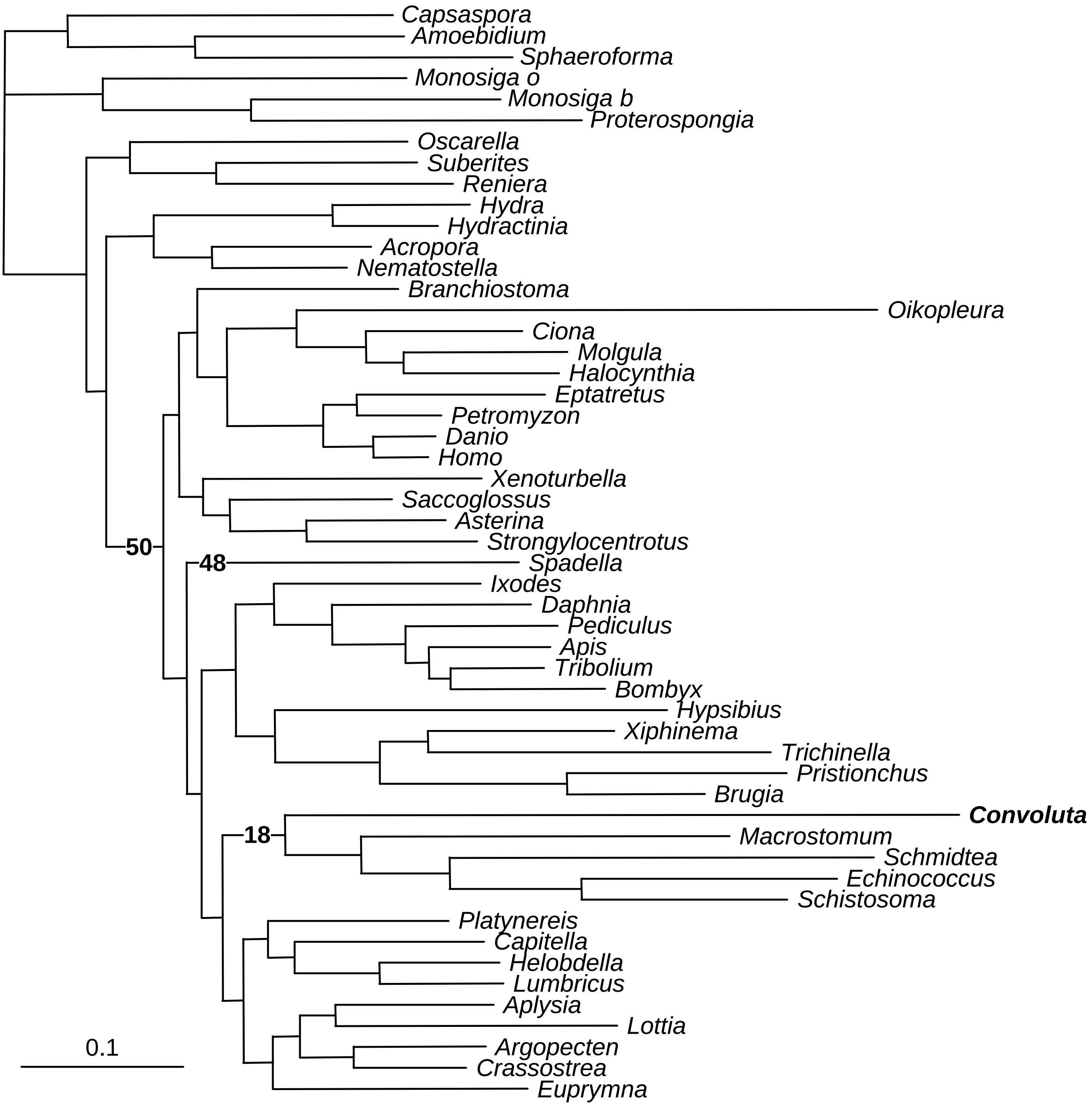

rank	item	obs	au	np	bp	pp	kh	sh	wkh	wsh
1	18	-55.9	0.974	0.917	0.915	1.000	0.959	1.000	0.946	1.000
2	48	55.9	0.084	0.027	0.027	5e-25	0.041	0.868	0.041	0.539
3	50	60.6	0.114	0.040	0.041	5e-27	0.054	0.847	0.054	0.666

Figure S3: Comparison of all possible placements of *Convoluta* within Holozoa. The possible positions of *Convoluta* were tested using the program CONSEL (Shimodaira & Hasegawa 2001) on the alignment of 11,959 positions with the WAG+Γ model. Among 99 possible positions, only three were not rejected by the AU test. The number of the topology indicated in second first column is reported on the topology to indicate the position of *Convoluta*.
