# Supplementary material for: Acoel Flatworms Are Not Platyhelminthes: Evidence from Phylogenomics
Source: PLoS One. 2007 Aug 8;2(8):e717. doi: 10.1371/journal.pone.0000717 (PMC1933604; doi:10.1371/journal.pone.0000717)
Supplement: Figure S6 — Bayesian tree inferred from 11,959 unambiguously aligned amino acid positions without Convoluta and the outgroup (Ichthyosporea, Choanoflagellata, Porifera and Cnidaria) using the CAT model. The robustness of the phylogenetic inference was estimated through 100 bootstrap replicates. Nodes supported by bootstrap values of 100% are denoted by black circles while lower values are given explicitly. The scale bar indicates the number of changes per site. (0.01 MB PDF) [file pone.0000717.s009.pdf]

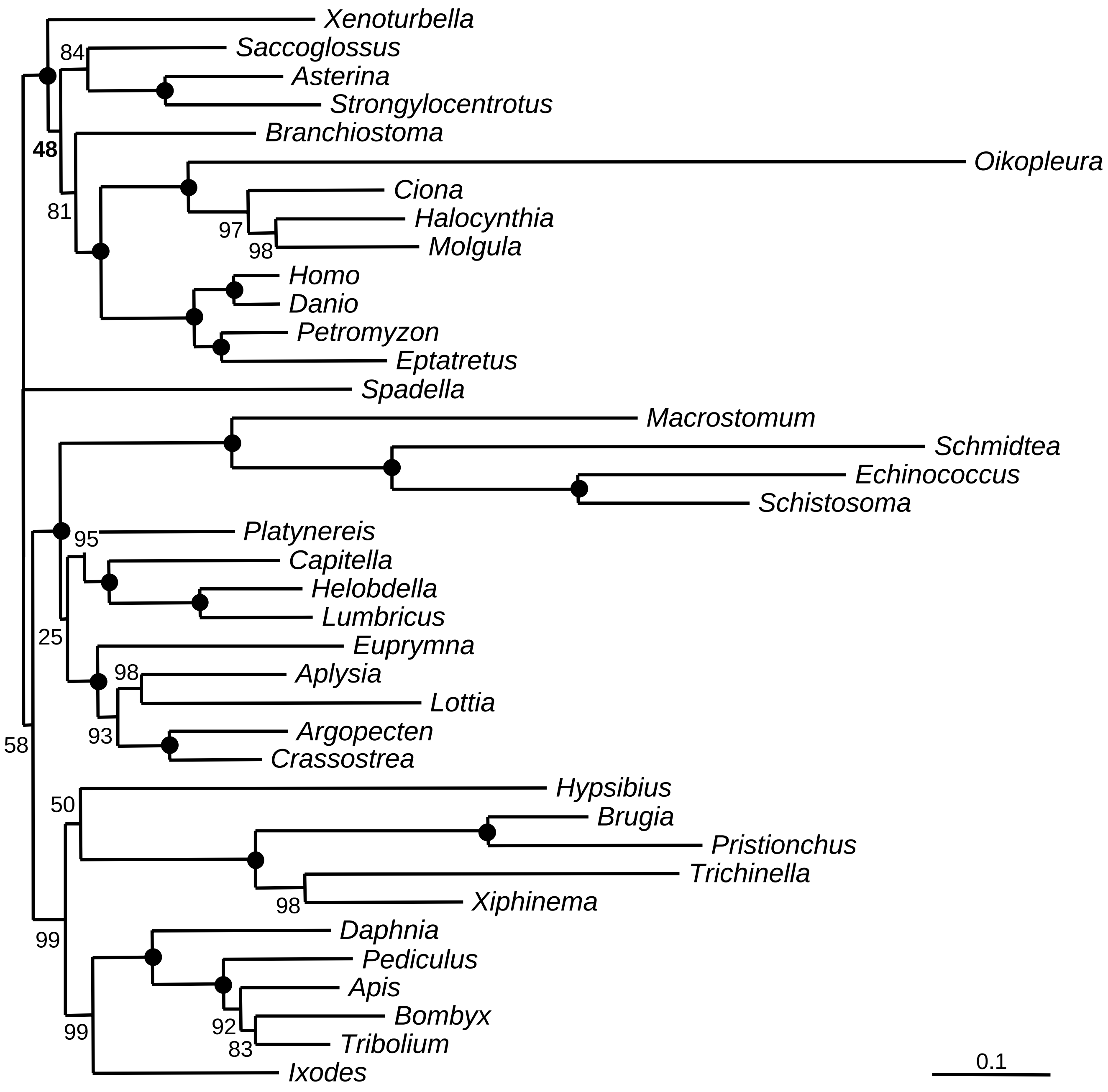

Figure S6: Bayesian tree inferred from 11,959 unambiguously aligned amino acid positions without *Convoluta* and the outgroup (Ichthyosporea, Choanoflagellata, Porifera and Cnidaria) using the CAT model. The robustness of the phylogenetic inference was estimated through 100 bootstrap replicates. Nodes supported by bootstrap values of 100% are denoted by black circles while lower values are given explicitly. The scale bar indicates the number of changes per site.
