# Supplementary material for: Acoel Flatworms Are Not Platyhelminthes: Evidence from Phylogenomics
Source: PLoS One. 2007 Aug 8;2(8):e717. doi: 10.1371/journal.pone.0000717 (PMC1933604; doi:10.1371/journal.pone.0000717)
Supplement: Figure S7 — Comparison of all possible placements of Convoluta within Holozoa when Platyhelminthes are removed. The possible positions of Convoluta were tested using the program CONSEL (Shimodaira & Hasegawa 2001) on the alignment of 11,959 positions with the WAG+Γ model. Among 91 possible positions, six were not rejected by the AU test. The number of the topology indicated in the second column is reported on the topology to indicate the position of Convoluta. (0.02 MB PDF) [file pone.0000717.s010.pdf]

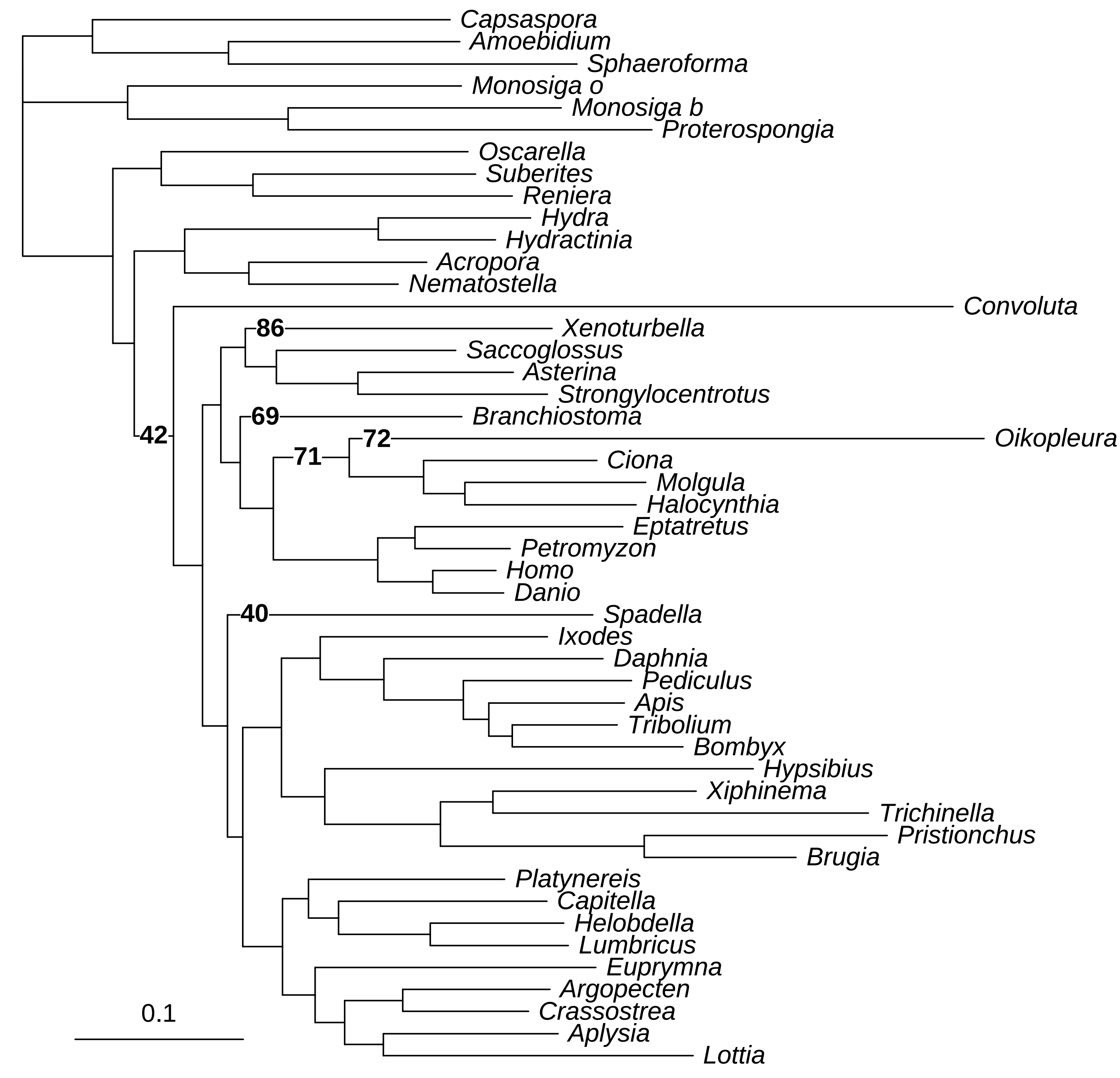

rank	item	obs	au	np		bp	pp	kh	sh	wkh	wsh
1	42	-4.0	0.809	0.429			0.420	0.981	0.566	1.000	0.566	1.000
2	40	4.0	0.647	0.358			0.366	0.019	0.434	0.996	0.434	0.994
4	86	30.2	0.173	0.047			0.048	8e-14	0.115	0.951	0.115	0.790
6	69	40.9	0.115	0.017			0.017	2e-18	0.060	0.913	0.060	0.607
7	72	47.2	0.213	0.111			0.113	3e-21	0.162	0.824	0.162	0.753
10	71	53.9	0.078	0.010			0.010	4e-24	0.064	0.832	0.064	0.615

Figure S7: Comparison of all possible placements of *Convoluta* within Holozoa when Platyhelminthes are removed. The possible positions of *Convoluta* were tested using the program CONSEL (Shimodaira & Hasegawa 2001) on the alignment of 11,959 positions with the WAG+Γ model. Among 91 possible positions, six were not rejected by the AU test. The number of the topology indicated in the second column is reported on the topology to indicate the position of *Convoluta*.
